# Leaky RAG Deficiency in Adult Patients with Impaired Antibody Production against Bacterial Polysaccharide Antigens

**DOI:** 10.1371/journal.pone.0133220

**Published:** 2015-07-17

**Authors:** Christoph B. Geier, Alexander Piller, Angela Linder, Kai M. T. Sauerwein, Martha M. Eibl, Hermann M. Wolf

**Affiliations:** 1 Immunology Outpatient Clinic, Vienna, Austria; 2 Biomedizinische ForschungsgmbH, Vienna, Austria; Pavillon Kirmisson, FRANCE

## Abstract

Loss of function mutations in the recombination activating genes RAG1 and RAG2 have been reported to cause a T^-^B^-^NK^+^ type of severe combined immunodeficiency. In addition identification of hypomorphic mutations in RAG1 and RAG2 has led to an expansion of the spectrum of disease to include Omenn syndrome, early onset autoimmunity, granuloma, chronic cytomegalovirus- or EBV-infection with expansion of gamma/delta T-cells, idiophatic CD4 lymphopenia and a phenotype resembling common variable immunodeficiency. Herein we describe a novel presentation of leaky RAG1 and RAG2 deficiency in two unrelated adult patients with impaired antibody production against bacterial polysaccharide antigens. Clinical manifestation included recurrent pneumonia, sinusitis, otitis media and in one patient recurrent cutaneous vasculitis. Both patients harbored a combination of a null mutation on one allele with a novel hypomorphic RAG1/2 mutation on the other allele. One of these novel mutations affected the start codon of RAG1 and resulted in an aberrant gene and protein expression. The second novel RAG2 mutation leads to a truncated RAG2 protein, lacking the C-terminus with intact core RAG2 and reduced VDJ recombination capacity as previously described in a mouse model. Both patients presented with severely decreased numbers of naïve CD4^+^ T cells and defective T independent IgG responses to bacterial polysaccharide antigens, while T cell-dependent IgG antibody formation e.g. after tetanus or TBEV vaccination was intact. In conclusion, hypomorphic mutations in genes responsible for SCID should be considered in adults with predominantly antibody deficiency.

## Introduction

The adaptive immune system is critically dependent on the diverse expression of B cell immunoglobulin receptor (BCR) and T cell receptor (TCR). [1] To obtain the necessary level of diversity, the recombination of the variable (V), diversity (D) and joining (J) segments that form these receptors is directed by recombination activating gene 1 (RAG1) and 2 (RAG2). RAG1 and RAG2 form the recombinase complex which binds and cleaves specific recombination signals that flank VDJ regions. [2,3] During lymphopoiesis, the levels of RAG1 and RAG2 are tightly regulated, with two major peaks of expression occurring during T cell development, first in the double negative stage during TCR β gene recombination, and subsequently in the double positive stage when the TCR α chain genes are rearranged. [4] During B-cell development, RAG1 and 2 are first expressed in pro-B cells during heavy chain loci rearrangement and once again in pre-B cells during light chain recombination. [5]

Defects in RAG1 and RAG2 are known to cause a T^-^B^-^NK^+^ form of severe combined immunodeficiency. Since the first description of RAG1 and RAG2 deficiency in patients with severe combined immunodeficiency by Schwarz et al. in 1996 [6], a pleiotropic spectrum of phenotypes associated with RAG1/2 deficiency has been described. The spectrum of the disease has expanded to include Omenn syndrome, early onset autoimmunity, granuloma, chronic cytomegalovirus or EBV infection with expansion of gamma/delta T-cells, idiophatic CD4 lymphopenia and a phenotype resembling common variable immunodeficiency. [7–22]

While patients with pronounced hypogammaglobulinemia and agammaglobulinemia are well known to be susceptible to bacterial infections, [23] a clinically relevant selective deficiency in antibody production can also lead to significant susceptibility to bacterial infections even in patients with normal levels of serum immunoglobulins.[24] The most frequent selective antibody deficiency with normal immunoglobulin serum levels is a defect in IgG antibody production against bacterial polysaccharides in the presence of normal IgG antibody production to T-dependent protein antigens. First described by Umetsu et al. in patients with IgG2-IgG4 subclass deficiency, impaired antibody production against bacterial polysaccharide antigens was later confirmed to occur even in patients with normal IgG subclass levels. [25] Although such a specific antibody deficiency is a well-recognized predominantly antibody deficiency [26], the genetic defect has only been characterized in a minor subset of patients. Hypomorphic mutations in BTK have been described as a cause of selective anti-polysaccharide antibody deficiency [27]. Furthermore impaired antibody production against bacterial polysaccharide antigens was found in CD21 deficiency, CD20 deficiency, JAK3 deficiency, partial trisomy 19q13, nuclear factor-κB (NF-κB) essential modulator (NEMO) deficiency, and chromosome 18p deletion syndrome with IgA deficiency. [28–32]

In the present study we describe two unrelated adult patients with impaired antibody production against bacterial polysaccharide antigens and a leaky RAG1/2 deficiency harboring a combination of a null mutation on one allele and a novel hypomorphic mutation on the other allele. One novel mutation affected the RAG1 start codon and resulted in an aberrant gene and protein expression of RAG1. The other novel mutation affected RAG2. This RAG2 mutation had been shown in a mouse model to lead to a truncated protein lacking the C-terminal part while leaving the core region intact.[33] Both patients presented with a severe deficiency in naïve CD4^+^ T-cells and a defective T-independent IgG response to bacterial polysaccharide antigens while production of T cell dependent IgG antibodies e.g. against tetanus toxoid or TBEV was intact.

## Materials and Methods

### Ethics statement

This study was conducted in accordance with the Declaration of Helsinki. The patients gave their written informed consent that anonymized data collected as part of the routine medical attendance (immunological analysis, flow cytometry analysis and genetic mutation analysis) could be included in a scientific publication. All results presented in this study were obtained as part of the routine medical attendance the patients received and no extra intervention for this study. The son of patient B was investigated for possible primary immunodeficiency on request of his mother, and written informed consent was given to present anonymized results of this investigation. All patient information contained in this study was anonymized and de-identified prior to analysis, and only anonymized and de-identified patient information is contained in this study. When we presented this non-interventional study to the appropriate ethics committee, the Ethics Committee of the City of Vienna, evaluation was refused, according to the legal regulations to be applied (§15a Abs. 3a Wiener Krankenanstaltengesetz) no ethics committee evaluation is required for this type of study. (S1 Supporting Information)

### Patient and control blood samples

Peripheral venous blood was collected in either untreated (for antibody and immunoglobulin determinations) or lithium-heparin- (for genetic analysis and cell function tests) or EDTA- (for flow cytometry) containing tubes from patients, family members and healthy adult blood donors that served as controls. All patients were treated at the Immunology Outpatient Clinic in Vienna.

### Flow cytometry

Peripheral blood was used to analyze lymphocyte subpopulations by multi-color flow cytometry using standard protocols. S1 Table shows the monoclonal fluorophore conjugated antibodies used. Data were analyzed on a FACSCalibur (Becton Dickinson; USA), using standard protocols and evaluated using CELLQuest software (Becton Dickinson; USA).

### Determination of serum immunoglobulins and antibodies

Serum concentrations of immunoglobulins and IgG subclasses were determined by standard laser nephelometry on a Siemens nephelometric analyzer (Siemens Healthcare; Germany) using reagents purchased from Siemens-Behring Division. IgG and IgM antibodies against bacterial and viral antigens were determined using commercially available enzyme-linked immunosorbent assay (ELISA) kits (IgG antibodies against tetanus and diphtheria toxoid, tick borne encephalitis (TBE) virus, Haemophilus influenza type b (Hib)) or an in-house produced ELISA (IgG and IgM antibodies against 23-valent pneumococcal capsular polysaccharide) as previously described.[34]

### DNA isolation and targeted resequencing

Genomic DNA was prepared from peripheral blood by spin column purification (QIAamp DNA Blood Mini Kit; QIAGEN, Germany). Targeted resequencing of 222 primary immunodeficiency genes listed in the 2011 IUIS expert committee report was performed for the two index patients. (S2 Table) Nextera Custom Enrichment kit was used according to standard protocols (Illumina, USA). Targeted DNA library was quantified and validated using Illumina Eco Realtime (Illumina; USA) and Agilent Bioanalyzer (Agilent Technologies; USA). The library was sequenced in a multiplex pool on a single (151 bp paired-end reads) Miseq flowcell. (Illumina, USA) Data analysis was performed using CLC Genomic Workbench (QIAGEN, Germany)

### Allele-specific PCR

The coding sequence of RAG1 and RAG2 was amplified using Phire Hot Start II DNA Polymerase (Thermo Fisher Scientific; USA). Allele-specific PCR was used to characterize RAG1 M1V and R737H alleles. Each allele (wild type and mutant form) was amplified separately with an allele-specific primer in combination with a general primer using Maxima Hot Start Taq DNA Polymerase (Thermo Fisher Scientific, USA). The resulting amplicons were purified and custom Sanger sequenced (Eurofins Genomic; Germany). Sequences were aligned to NM_000448.2 RAG1 and NM_001243785.1 RAG2, using CLC Genomic Workbench (QIAGEN, Germany)

### T-cell CDR3 Vβ Spectratyping

Examination of the TCR Vβ repertoire of CD3^+^ T-cells was performed by spectratyping analysis as described previously. [35] The primer sequences used for amplification are shown in S3 Table. Fragment length analysis sequences were acquired using an ABI 3130 XL Sequencer (ABI Applied Biosystems; USA) and analyzed using Peak Scanner2 software (ABI Applied Biosystems; USA).

### Cloning of RAG1

Two constructs were generated: wild type RAG1 (NM_000448.2) and mutant M1V RAG1. The full-length RAG1 ORF was synthesized (Life Technologies; USA) and the constructs were subcloned into the pcDNA3.3 Vector System. (Life Technologies, USA) The plasmids were transfected into Freestyle HEK 293 cells (Life Technologies, USA) to express wild type RAG1 or mutant RAG1. Mock transfected cells were used as a control. Cells were cultured at 37°C in a humidified atmosphere with 5% CO_2_ in complete FreeStyle 293 Expression medium (Life Technologies, USA). Samples were harvested at 96 hours post transfection.

### RNA isolation, reverse transcription and quantitative Real-Time PCR (qRT-PCR)

RNA was extracted from frozen RAG1 transfected HEK 293 cells using RNeasy Mini Kit (Qiagen; USA). RNA quality was assessed using Agilent RNA 6000 Nano Kit (Agilent Technologies, USA) on a 2100 Bioanalyzer Instrument (Agilent Technologies, USA), and only RNA with an RNA Integrity Number (RIN) higher than 9 was used for subsequent analysis. Equal amounts of RNA were reversely transcribed into cDNA using the SuperScript VILO cDNA Synthesis Kit (Life Technologies, USA) following standard protocols. qRT-PCR was performed as previously described.[36] In short, 10ng of cDNA was amplified using an Eco Real-Time PCR System (Illumina, USA) with KAPA SYBR FAST SuperMix (PEQLAB). The ΔΔCT-method was used to calculate relative gene expression. Expression of the housekeeping gene HPRT served as an internal standard. RAG1 and HPRT primer sequences are shown in S3 Table.

### In-vitro immunoglobulin secretion and proliferation

Peripheral blood mononuclear cells (PBMCs) of patient A and controls were stimulated with Epstein-Barr virus (EBV) using the supernatant from B 95–8marmoset cell line (ATCC, Rockville, MD) as previously described ([32]). 1*10^6^ cells per milliliter were incubated for 8 days in complete RPMI 1640 medium supplemented with 10% heat-inactivated fetal calf serum, 2mM L-glutamine, 100 IU/ml penicillin, and 100μg/ml streptomycin (Gibco, Paisley, UK) at 37°C in the presence of 5% CO_2_. After the incubation period supernatants were harvested and an in-house Enzyme-linked Immunosorbent Assay was performed to determine IgG and IgM level as described previously.[32] Results are expressed as mean ± standard deviation of triplicate measurements. PBMC proliferation was assessed by ^3^H-Thymidin incorporation using standard protocols as described previously.[37]

### Western Blot

Wild type and mutant RAG1-transfected HEK 293 cells were lysed for 30 min in ice-cold RIPA lysis buffer system (Santa Cruz Biotechnology, USA), and insoluble material was removed by centrifugation (16,000 x g, 10 min, 4°C). 20μg of protein were resolved in 8%SDS-polyacrylamide gel electrophoresis (SDS-PAGE) buffer, electrotransferred onto a polyvinylidene difluoride membrane (Immobilon-P; Millipore), and immunoblotted with anti-RAG1 antibody (H-300) (Santa Cruz Biotechnology Inc; USA). Detection was performed using the SuperSignal West Pico ECL detection system (Thermo Scientific; USA).

## Results

### Case Report

**Fig 1:**
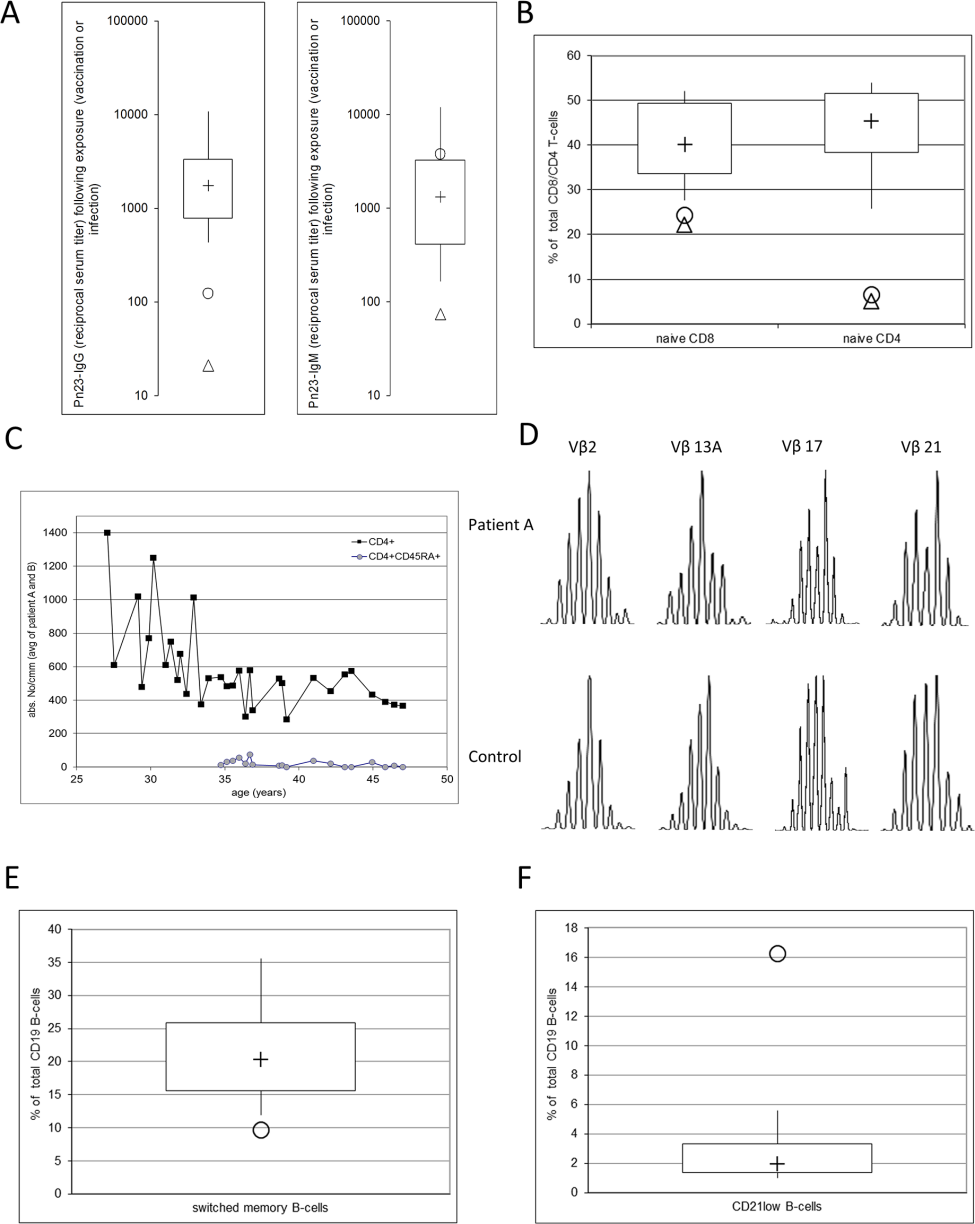
Immunological characteristics of patients with hypomorphic RAG deficiency and impaired antibody production against bacterial polysaccharide antigens. Patient A…circles; patient B…triangles 22 (A) Impaired antibody production against bacterial polysaccharide antigens in RAG-deficient patients. Box-plots represent pn23-antibodies in 41 healthy individuals (B) Numbers of CD4^+^CD45RA^+^ (naïve CD4) and CD8^+^CD45RA^+^CD62L^+^ (naïve CD8) T cells given as percentage of total CD8- or CD4-positive T cells. Box-plots depict 50 healthy individuals (C) Kinetic of the total count of CD4^+^ T cells and naïve CD4^+^CD45RA^+^ T cell depicted as average of both patients (D) Representative example of the distribution of TCR Vbeta genes 16, 17, 18, 22 (E) Numbers of switched memory B cells given as percentage of total CD19 B cells. Box-plots depict 50 healthy individuals (F) Numbers of CD21^low^ B cells given as percentage of total CD19 B cells. Box-plots depict 50 healthy individuals (median,+; box, IQR; whiskers, q5-q95).

Patient A is a woman who was first investigated for primary immunodeficiency at the age of 27 years because of a history of undue susceptibility to infections and known hypogammaglobulinemia first diagnosed at the age of 13 years. She was born to unrelated parents of Austrian origin, her brother and mother are healthy, her father died due to coronary heart disease. Recurrent bacterial infections started at the age of seven with recurrent pneumonia and bronchitis, recurrent sinusitis and otitis media. At the age of eight she was hospitalized because of vasculitis of the skin of both legs, with recurrence at 13 and 36 years of age at which time a biopsy was performed and the histology revealed leukocytoclastic vasculitis. At the age of ten chronic interstitial pneumonia was diagnosed, an open biopsy of the lung revealed diffuse panbronchiolitis. Chronic obstructive airway disease with recurrent bacterial infections of the upper and lower airways and hospitalizations due to infection-associated exazerbation of the lung problems constituted the clinical picture since the age of 13 years. Streptococcus pneumoniae was repeatedly identified in sputum cultures and middle ear effusions during episodes of upper and lower respiratory tract infections. IVIG substitution therapy was started at the age of 20 years because of hypogammaglobulinemia with a pretreatment serum IgG level of 393mg/dl and levels of IgA and IgM within the normal range. A treatment-free interval of 6 months at the age of 28 revealed mild hypogammaglobulinemia (serum IgG, mg/dl [normal range]: 697 [790–1700]) IgG1-subclass deficiency (serum IgG1, mg/dl [normal range]: 303 [500–880], normal levels of IgA, IgM, IgG2-4 and low pneumococcal IgG-antibodies despite the fact that recurrent pneumococcal infections had led to very high IgM-antibody titers (Fig 1A). IgG-anti-polysaccharide antibody deficiency was diagnosed and the rapid reappearance of pulmonary infections required resumption of two-weekly IVIG substitution therapy. While regular IVIG substitution therapy led to a long-term improvement of the susceptibility to pulmonary and middle ear infections, the clinical picture was finally dominated by obstructive lung problems with decreasing lung function that made a 24-hour oxygen-therapy necessary and ultimately led to the demise of the 48 year old patient.

Patient B is a 41-year-old woman living in the rural province of upper Austria who was first investigated for primary immunodeficiency at the age of 35 years shortly after her first pneumonia with a history of recurrent bacterial bronchitis during the last four years in the absence of known risk factors such as allergies or smoking. She has two healthy children, a son of nine years and a daughter of six years. IgA-, IgM- and IgG2-IgG3-IgG4 subclass deficiency (serum IgG, mg/dl [normal range]: 940 [790–1700]; serum IgG2, mg/dl [normal range]: 51 [150–600]; serum IgG3, mg/dl [normal range]: 5 [20–100]; serum IgG4, mg/dl [normal range]: <7 [8–120]; serum IgA, mg/dl [normal range]: <6 [76–450]; serum IgM, mg/dl [normal range]:29 [90–350]) with normal levels of IgG1 (data not shown) and antibody deficiency against bacterial polysaccharides was diagnosed. Regular IVIG substitution therapy was initiated at the age of 35 years, which led to normalization of the susceptibility to infections. Following the birth of her third child and a substantial increase in the patient´s body weight another pneumonia occurred, followed by recurrent sinusitis and obstructive bronchitis with limited clinical improvement despite an increase in IVIG dosage and sinus surgery.

### Hypomorphic mutations in RAG1 and RAG2

**Fig 2:**
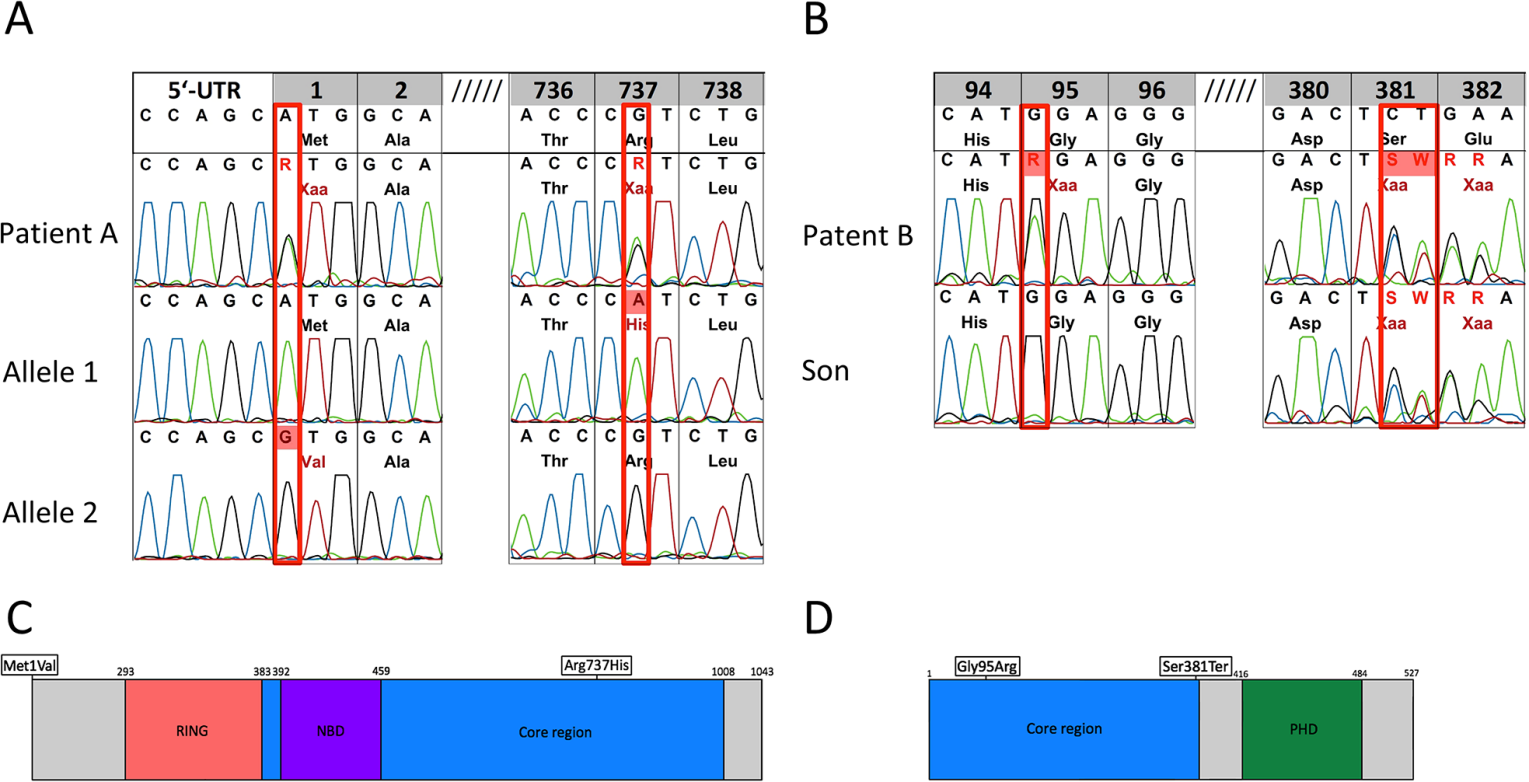
RAG1/2 Mutation analysis of genomic DNA from peripheral blood. (A) Patient A is compound heterozygous in RAG1, c.1 A>G, p.M1V; c.2322 G>A, p.R737H. Allele-specific PCR was used to characterize RAG1 M1V and R737H alleles. (B) Patient B is compound heterozygous in RAG2, c1347-8delCT, pSer381Terfs*1; c488G>A, pGlu95R. Mutation analysis of the son of patient B identified the compound heterozygosity of the RAG2 missense mutations. (C) Schematic depiction of RAG1 (D) Schematic depiction of RAG2. Mutations are indicated by rectangles.

To determine the underlying disease-causing genetic defect we screened both patients for mutations in the primary immunodeficiency genes listed in the 2011 IUIS expert committee report. [26] Targeted resequencing of 222 primary immunodeficiency genes and potential candidate genes was performed for the two index patients, genes sequenced are listed in S2 Table. Data analysis and filtering strategy is depicted in S1 Fig. In both patients no disease-causing mutations were identified in genes known to be responsible for predominantly antibody deficiency syndromes. In contrast, genetic testing in patient A revealed a compound heterozygous mutation in RAG1 (c.125 A>G, p.M1V; c.2334 G>A, p.R737H) (Fig 2A and 2C). The c2334 G>A transition in exon 2 resulted in a missense mutation which led to an arginine to histidine substitution at position 737 in the active core of RAG1. This mutation has been previously reported by A. Villa et al. in a patient with Omenn syndrome. In this study the recombination efficiency of mutant RAG1 was examined and the R737H substitution led to a total lack in recombinase activity. [38]

**Fig 3:**
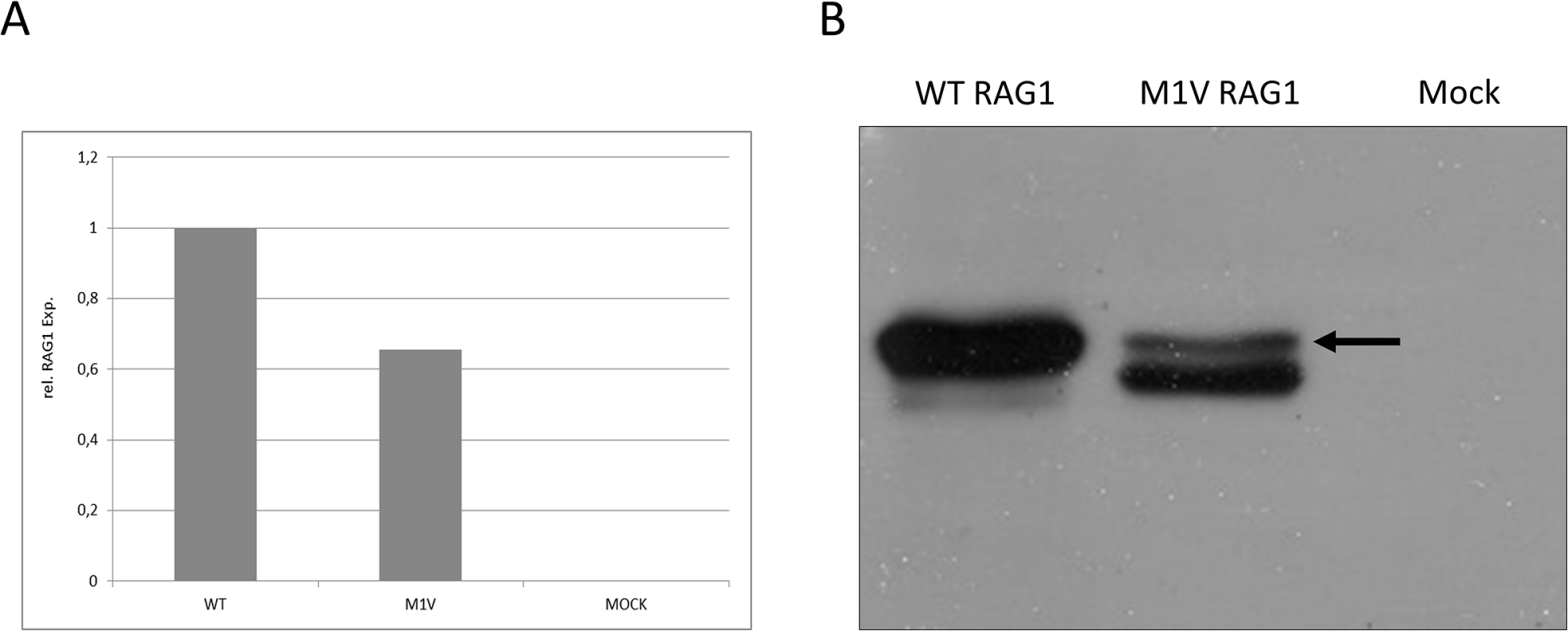
Molecular characterization of novel M1V RAG1 mutation. (A) Gene expression of RAG1 in HEK293 cells transfected with wild-type RAG1 and M1V RAG1 mutant by quantitative real time PCR. Cells transfected with mock vector served as a control. Wild type expression was normalized to 1 and percentage of RAG1 M1V gene expression was calculated. (B) Protein expression of RAG1 in HEK293 cells transfected with wild-type RAG1 and M1V RAG1 mutant by western blot. Cells transfected with mock vector served as a control. The arrow indicates RAG1 protein as identified through its molecular weight of 130kD.

The RAG1 mutation found in the second allele of patient A (c.125 A>G) was a novel mutation which affected the start codon of RAG1 (ATG>GTG). We hypothesized that this is a hypomorphic mutation which could result in an aberrant gene expression of RAG1. RAG1 expression was assessed by using HEK 293 cells transduced with either wild-type or M1V mutant RAG1. Mutant RAG1 mRNA expression was moderately, by approximately 35%, reduced as compared to wild type RAG1 gene expression. (Fig 3A). In contrast, western blot analysis revealed a profound defect in RAG1 protein expression in cells transfected with the mutant RAG1 gene (Fig 3B).

Patient B harbored two mutations in RAG2 (c1342-3delCT, pSer381Terfs*1; c683G>A, pGly95Arg) that were shown to be compound heterozygous by mutation analysis of patient´s son (Fig 2B and 2D). The c683G>A missense mutation changes glycine 95 into arginine and has previously been reported in a patient with Omenn syndrome. [39] G95 is conserved in the RAG2 gene of all known species and is located in a kelch motif in the enzymatically active core. Functional studies revealed an ablation of in vitro and in vivo recombination when RAG2 G95R mutant was tested.

The RAG2 mutation on the second allele of patient B (c.1342-3delCT) is a novel mutation never described before in men. We refrained from further investigating the molecular consequences of this mutation because a mouse model harboring a mutation, with similar consequences for Rag2 protein function, was previously described by Akamatsu and colleagues, [33] where it was shown that the mutation results in a frameshift and stop codon at position 381 (pSer381Terfs*1). The mouse model expressed a truncated RAG2 protein lacking the C-terminal 145 amino acids while leaving the catalytic core region of RAG2 intact. Mice expressing the mutant, truncated “core RAG2” (1–383) retained significant in vivo function of VDJ recombination but displayed a reduction in total numbers of B and T cells, presumably due to impaired lymphocyte development at the progenitor stage associated with reduced VDJ recombination, but cell numbers that were sufficient to prevent the development of a SCID phenotype.

### Leaky RAG deficiency is associated with a severe reduction in naïve CD4 T cells

In both patients lymphocyte counts and numbers of CD4^+^, CD3^+^ and CD56^+^ lymphocytes as well as numbers of Treg cells (CD4^+^CD25^+^CD127^low^) were within the normal range, while iNKT cells (CD3^+^TCRVα24^+^TCRVβ11^+^lymphocytes) were undetectable (data not shown) However, a more detailed characterization of the CD4^+^ T cells showed a severe reduction in naïve CD4^+^ cells (CD4^+^CD45RA^+^) in both patients (Fig 1B), while both CD4^+^ memory subsets (central memory CD4^+^CD45RA^-^CD62L^+^ and effector memory CD4^+^CD45RA^-^CD62L^-^) were present in normal numbers (data not shown). The total count of CD4^+^ T cells decreased over-time in both patients. (Fig 1C) In contrast to the severe reduction of naïve CD4^+^ T cells, naïve CD8^+^ T cells (CD8^+^CD45RA^+^CD62L^+^) were only slightly reduced (Fig 1B). The number of CD8^+^ T cell subsets (CD8^+^CD45RA^+^CD62L^-^ effector cells, CD8^+^CD45RA^-^CD62L^-^ effector memory cells, and CD8^+^CD45RA^-^CD62L^+^ central memory cells) were comparable to healthy controls in patient A, while the number of CD8^+^ effector memory cells was increased in patient B (% of CD8^+^ lymphocytes [normal range] at age 41: 36.8 (7.4–24.8). Lymphoproliferative responses to mitogenic stimulation (PHA, ConA, PWM) were within the normal range in both patients (data not shown).

To further investigate a possible role of decreased RAG activity on T cell diversity, given that the number of naïve CD4^+^ T-cells emigrating from the thymus was reduced, we analyzed T cell receptor of diversity CD3^+^ T-cell in patient A. TCR spectratyping analysis revealed a diverse distribution of all 23 TCR Vbeta genes, thus giving no indication for a restricted TCR repertoire in this patient. Fig 1D shows the distribution of 4 frequently used TCR Vbeta genes. (Fig 1D) S2 Fig shows the distribution of TCR Vbeta genes 1–23.

### Impaired antibody production against bacterial polysaccharide antigens in adult patients with leaky RAG1/2 deficiency

The antibody response to a range of T-cell dependent and T cell independent antigens was investigated and the results indicated a lack of IgG responsiveness to T-independent bacterial polysaccharide antigens in both patients. Patient B displayed a markedly reduced IgG and IgM response to vaccination with 23-valent unconjugated pneumococcal (Pneumo 23 “Merieux”) and four-valent unconjugated meningococcal (Mencevax)-vaccine (reciprocal serum titer against ACW_135_Y meningococcal capsular polysaccharide: before vaccination, IgG <20, IgM <20; after vaccination, IgG <20, IgM <20; normal response in vaccinated healthy adults, IgG ≥100, IgM ≥100). Patient A displayed a poor Streptococcus pneumonia anti-capsular IgG response despite the fact that repeated culture proven pneumococcal infections led to high titers of IgM antibodies against these antigens (Fig 1A), resulting in a ratio of IgG- to IgM-Pn23-antibody serum titer of 0.03, well below the values observed in healthy adults either before or after Pn23 vaccination (ratio of IgG- to IgM-Pn23-antibody serum titer in 41 healthy adults [median, q5-q95]: before Pn23 vaccination, 2.00, 0.35–22.77; six-eight weeks after Pn23 vaccination¸ 0.95, 0.17–45.89). In contrast, IgG antibody responses to protein antigens such as tick-borne encephalitis virus (TBEV) vaccine were normal in both patients. (Serum TBEV IgG, ELISA, VIEU/ml: patient A, before vaccination 213, after vaccination 1609; patient B, before vaccination 51, after vaccination 1694; normal vaccination response in healthy adults ≥310). Furthermore, serum IgG antibodies against tetanus toxoid, diphtheria toxoid, polio I,II,III could be examined in patient B after vaccination and before IVIG replacement therapy and were found to be within the normal range (data not shown). In addition, serum IgG antibodies against measles, mumps, rubella and VZV were detectable in patient B before IVIG replacement therapy (data not shown).

### B cell differentiation is impaired in leaky RAG1/2 deficiency

Patient A displayed normal levels of circulating B cells (CD19- and CD20-positive lymphocytes as determined by flow cytometry) but we observed a shift in peripheral blood B cell subsets. Naïve CD27^-^IgD^+^ and MZ-like CD27^+^IgD^+^ B cells were present in normal numbers, while numbers of IgD^-^CD27^+^ switched memory B cells (9,7% of total B-cells) were decreased. (Fig 1E) Furthermore we observed an expansion of CD21^low^ B-cells, as numbers of this B cell subset were increased to 16,3% of total B-cells as compared to 1,95% in healthy adult donors (median of N = 50) (Fig 1F). CD21^low^ B cells are proposed to be an exhausted, anergic B-cell population with possible autoreactive capacities.

In patient B B cell numbers (identified as CD19-positive lymphocytes by flow cytometry) were significantly reduced but detectable upon first examination (CD19^+^ lymphocytes at age 35 [normal range]: % of lymphocytes, 2 [7–23]; absolute number/μl, 21 [71–549]) and decreased continuously over time until they were completely undetectable at age 41.

### Decreased in-vitro IgG production by EBV-transformed B cells with leaky RAG1/2 deficiency

To assess the capacity of in-vitro immunoglobulin production in patient A with hypomorphic RAG1 mutation, peripheral blood mononuclear cells (PBMCs) were simulated with Epstein-Barr virus (EBV). IgG in-vitro production was markedly reduced in patient A, compared to healthy controls. In contrast IgM in vitro levels were was comparable to the controls (Table 1) Further we analyzed proliferation response following EBV transformation, there was no significant reduction observed between patient A and healthy blood donors. (Table 1)

**Table 1:** In vitro immunoglobulin production and ^3^H-Thymidin incorporation of PBMCs and lymphoblastoid cells.

immunoglobulin production							
in vitro (ng/ml)							
		control			patient		
		mean		SD	mean		SD
unstimulated	IgG	89	±	37	n.a.		
	IgM	103	±	53	n.a.		
EBV transformed	IgG	6388	±	4239	887	±	76
	IgM	24078	±	7946	38127	±	1805
^3^H Thymidin incorporation							
dpm							
unstimulated		1920	±	783	n.a.		
EBV transformed		75979	±	2795	90914	±	305

Unstimulated peripheral blood mononuclear cells and EBV-transformed lymphoblastoid cells were incubated for 8 days and the supernatant IgM/G concentration was determined by ELISA. Additionally the ^3^H-thymidin incorporation was determined. Results are expressed as mean and standard deviation (control unstimulated IgM/G: n = 9; control EBV-transformed IgM/G: n = 3; patient EBV-transformed IgM/G: n = 1 (triplicate); control unstimulated ^3^H-thymidin incorporation: n = 9; control EBV-transformed ^3^H-thymidin incorporation: n = 1 (triplicate); patient EBV-transformed ^3^H-thymidin incorporation: n = 1 (triplicate)).

## Discussion

We report herein a novel presentation of leaky RAG1/RAG2 deficiency which is associated with selective IgG antibody deficiency and massively decreased numbers of naïve CD4^+^ T-cells. Both adult patients presented with intact production of T-cell dependent IgG antibodies but a defective T-independent IgG response to bacterial polysaccharide antigens. In patient B defective IgG antibody response to bacterial polysaccharide could be demonstrated by measurement of post-vaccination antibody response. In patient A however, it was not possible to examine IgG response after pneumococcal vaccination before the initiation of immunoglobulin replacement therapy providing substantial levels of IgG antibodies against 23-vqlent pneumococcal polysaccharides (Pn23). However, despite recurrent culture-proven pneumococcal infections such as pneumonia IgG-Pn23-antibodies were low (representing a mix of IVIG-derived and patient-derived antibodies), while IgM-Pn23-antibodies (entirely derived from the patient) were very high, resulting in a ratio of IgG- to IgM-Pn23-antibody serum titer that was well below the minimal value observed in our laboratory in healthy adults either before or after Pn23 vaccination. The patients described experienced recurrent pneumonia, sinusitis, otitis media and recurrent cutaneous vasculitis in one patient. While patient A´s clinical symptoms started in childhood, patient B presented in her fourth decade of life for the first time with a relative short history of disease. In both patients IVIG substitution corrected most of the susceptibility to infections.

Using a novel strategy of targeted resequencing a multitude of known primary immunodeficiencies genes in patients with predominantly antibody deficiency we identified compound heterozygous mutations in RAG1 or RAG2 in the two patients described. Delayed onset and relatively mild clinical manifestations were consistent with a residual function of the described novel hypomorphic RAG mutations. Patient A displayed a well described R737H substitution in one allele of RAG1. A. Villa and colleagues previously reported this mutation in a homozygous form in a patient with Omenn-syndrome and found that this is a null mutation associates with a complete lack of recombination activity.[38] Patient A however harbored a novel M1V mutation on the other RAG1 allele that affected the initiation codon of RAG1 (ATG>GTG). Subsequent characterization of the novel M1V mutation revealed a reduced but detectable Rag1 protein expression. Residual expression of Rag1 might explain the relatively mild clinical phenotype. Although the compound heterozygous combination of one null RAG1 allele and one hypomorphic allele results in a severe reduction of total RAG1 protein, this aberrant expression might be sufficient enough to prevent the development of a SCID phenotype.

In patient B we identified compound heterozygous mutations in RAG2. Analogous to patient A one mutation was a null RAG2 mutation as described in the literature. [39] The mutation on the second allele was a novel frameshift mutation that, resulting in a truncated RAG2 protein with an intact catalytic core region. Although not required for the basic biochemistry of V(D)J recombination, the non-catalytic region of RAG1/RAG2 is conserved throughout evolution. The C terminus of RAG2 is predicted to contain a plant homeodomain (PHD), a motif required for chromatin protein interaction. Akamatsu Y. et al. generated a transgenic mouse model with an analogous mutation resulting in a truncated RAG2 with intact core region. [33] These transgenic mice displayed a phenotype similar to patient B. The overall number of peripheral T-cells was unaffected however mice exhibit a partial block in T-cell development. These data are consistent with our finding that levels of circulating naïve CD4^+^ T-cells were severely decreased in the RAG2-deficient patient as well. Although residual RAG1/2 expression/activity is sufficient to prevent the development of a SCID phenotype, impairment of lymphoid development may result from altered V(D)J recombination or decreased cell proliferation. As TCR spectratyping in patient A revealed a diverse repertoire one might speculate that the hypomorphic RAG1/2 mutations lead to a proliferation disadvantage. Cell cycle-regulated periodic destruction of RAG2 at the G1-to-S transition is induced through the phosphorylation of Thr-490 located in the C-Terminus that is deleted in patient B. [40] Such a dysregulation might result in aberrant expression of RAG2 during S phase and thus lead to decreased cell proliferation and/or increased cell death. In individuals younger than 20 years the bulk of naïve T cells is sustained primarily from thymic output, whereas proliferation within the naïve T cell population dominates in older individuals.[41] These findings are consistent with our findings that in leaky RAG deficiency a continuous decrease of absolute numbers of CD4^+^ T-cells over time was observed. (Fig 1C) Patient B displayed a significant B-cell lymphopenia, and a comparable impairment in B cell differentiation was observed in core RAG2-deficient mice, as in this animal model mature circulating B cell numbers were severely reduced. Patient A exhibited a moderate B cell differentiation defect with a pronounced expansion of circulating CD21^low^ B-cells and decreased numbers of circulating switched memory B cells. This decrease in the percentage of switched memory B cells reflects the hypogammaglobulinemia and selective IgG antibody deficiency found in the patient and could also be responsible for the observed impairment in IgG production by patient A´s EBV-transformed B cells, although an additional effect of the patient´s RAG1 deficiency on immunoglobulin class switching cannot be ruled out.[42] Expansion of CD21low B cells is predominately found in HIV infected individuals and in a specific subgroup of CVID patients[43,44]. Recent findings suggest an anergic status of CD21^low^ B cells in CVID associated with a defect in calcium-dependent BCR-activation. [45] Further reports have demonstrated an increased proportion of autoreactive B cells among CD21^low^ B cells. [46] As patient A experienced episodes of leukocytoclastic vasculitis one might speculate that an increased expansion of autoreactive CD21low B cells might be implicated in the pathophysiology of her disease complications.

In both patients defects in B-cell function and differentiation were observed. A selective impairment in IgG antibody production against a certain type of antigen such as bacterial polysaccharides could be explained by a restricted B-cell repertoire, as has been shown in CD20 deficient patients. [30] It is feasible that a mutation in RAG leads to such a repertoire restriction, as V(D)J recombination is crucial to obtain BCR diversity. In contrast defects in cell-mediated immunity were limited to a decrease in naïve CD4^+^ T-cells. No restriction in TCR diversity was observed. The results indicate that hypomorphic RAG mutations have more impact on humoral then on cell-mediated immune responses. Which is consistent with previous findings in mice overexpressing dominant negative RAG1 that led to impaired B-cell but intact T- cell development.[47]

Genetic defects in RAG1 and RAG2 are known to impair V(D)J recombination, thereby causing T^-^B^-^NK^+^ severe combined immunodeficiency. However, in the last decade additional patients with RAG mutations were identified that present with a various degree of immune dysregulation and clinical manifestation. Most previous reports describing the different clinical phenotypes of RAG deficiency presented pediatric patients with late onset of combined immunodeficiency characterized by CD4 lymphopenia and/or impaired T cell function and hypo- or agammaglobulinemia with defective B cell differentiation [7,9,10,13–19] or early onset autoimmunity and granulomatous disease [8–11,15,16,20]. Few adult patients with leaky RAG deficiency have been reported, Abraham et al described 38-year-old man with skin rash and severe pan-T-cell lymphopenia rash but no susceptibility to infections or impaired antibody responses. A heterozygous frameshift mutation in RAG1 was found that was also present in his healthy father [21]. Recently, Buchbinder et al reported two unrelated adolescent patients with opportunistic infections, persistent lymphopenia and an early age of first clinical symptoms (recurrent bacterial sinopulmonary infections, viral infections, autoimmune disease) who exhibited compound heterozygous mutations in RAG1 and a prior clinical diagnosis of CVID made at the age of five years (patient 1) and 14 years (patient 2) of age [22]. The phenotype reported in our patients (adult RAG-deficient patients with a predominant clinical picture of antibody deficiency) confirms and extends the already reported spectrum of disease associated with RAG deficiency. In addition to a pronounced susceptibility to bacterial infections of the respiratory tract one of our patients showed episodes of cutaneous vasculitis reminiscent of the patient described by Sharapova et al.[16] In contrast to previous patients, in our adult patients the leaky RAG-deficient phenotype was dominated clinically and immunologically by an impairment in antibody production. Thus our findings contribute to the known complexity of divergent genotype-phenotype manifestations of RAG mutations and underline the importance of testing for hypomorphic SCID mutations even in adult patients with predominantly antibody deficiencies. If predominantly antibody deficiency is associated with a hypomorphic SCID mutation the disorder is likely to be more complex as compared to patients with other defects leading to a deficiency of humoral immunity. Given that clinical manifestation and/or diagnosis can occur in adulthood, it will have to be decided whether alternative treatment such as HSCT will improve long-term prognosis as compared to immunoglobulin replacement alone. Further studies are needed to improve survival and long-term quality of life in other adult patients with a comparable clinical and immunological phenotype.

## Supporting Information

S1 Fig Next generation sequencing filtering strategy. (TIF)

S2 Fig Distribution of TCR Vbeta genes 1–23. (TIF)

S1 Supporting Information Ethics statement of the Ethics Committee of the City of Vienna. (PDF)

S1 Table Monoclonal fluorophore conjugated antibodies for flow cytometry. (DOCX)

S2 Table Primary immunodeficiency genes sequenced. (DOCX)

S3 Table Primer sequences for T-cell CDR3 Vβ Spectratyping and RAG1. (DOCX)
