# Supplementary figures and images for: Leaky RAG Deficiency in Adult Patients with Impaired Antibody Production against Bacterial Polysaccharide Antigens
Source: PLoS One. 2015 Jul 17;10(7):e0133220. doi: 10.1371/journal.pone.0133220 (PMC4506145; doi:10.1371/journal.pone.0133220)

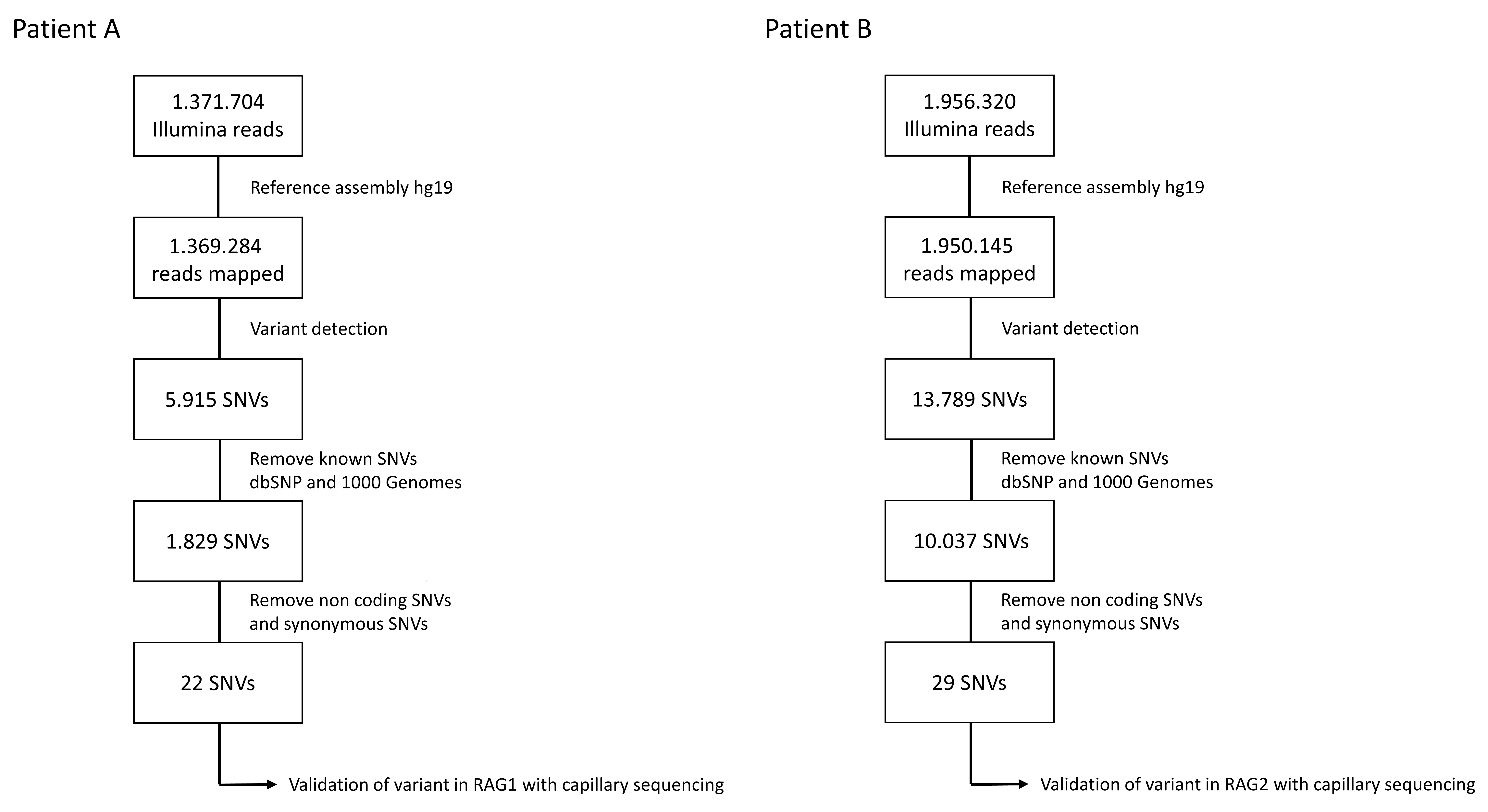

Supplement: S1 Fig — (TIF) [file pone.0133220.s001.tif]

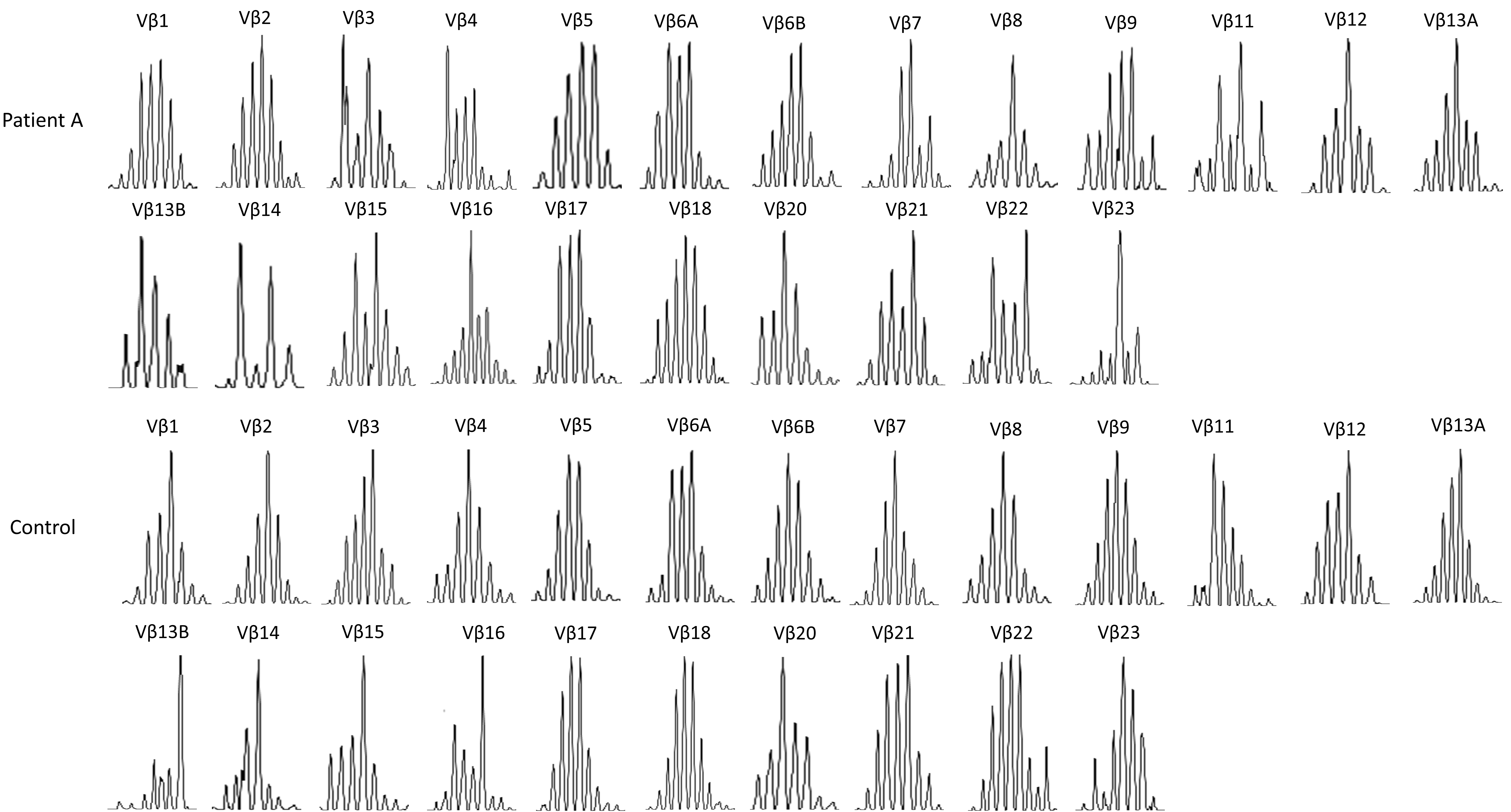

Supplement: S2 Fig — (TIF) [file pone.0133220.s002.tif]
