# Supplementary material for: Leaky RAG Deficiency in Adult Patients with Impaired Antibody Production against Bacterial Polysaccharide Antigens
Source: PLoS One. 2015 Jul 17;10(7):e0133220. doi: 10.1371/journal.pone.0133220 (PMC4506145; doi:10.1371/journal.pone.0133220)
Supplement: S1 Supporting Information — (PDF) [file pone.0133220.s003.pdf]

MAGISTRAT DER STADT WIEN
Magistratsabteilung 15 – Gesundheitsdienst der Stadt Wien
Ethikkommission der Stadt Wien
1030 Wien, Thomas-Klestil-Platz 8, Town Town 1. Stock, CB 12.103
Zugang: 1030 Wien, Schnirchgasse 12, Stiege 2, CB 12.103
Telefon: 40 00*, Fax: 40 00-99-877 54, E-Mail: ethikkommission@ma15.wien.gv.at
DVR: 0000191

EK_14_259_VK_NZ Wien, 11. Dezember 2014

Immunologische Tagesklinik
z.Hd. Herrn Univ.-Doz. Dr. Hermann Wolf
Schwarzspanierstrasse 15
1090 Wien

Sehr geehrter Herr Univ.-Doz. Dr. Wolf!

Bezugnehmend zu Ihrer Anfrage vom 2. Dezember 2014 zur Studie mit dem Titel:

„Leaky RAG deficiency in adult patients with selective polysaccharide antibody deficiency“

erlaube ich mir, auf den §15a Abs. 3a des Wiener Krankenanstaltengesetzes hinzuweisen.

Demnach vor der Durchführung angewandter medizinischer Forschung und von Pflegeforschungsprojekten und der Anwendung neuer Pflege- und Behandlungskonzepte und neuer Pflege- und Behandlungsmethoden eine Ethikkommission befasst werden kann!

Der Gesetzgeber schafft hierbei eine sogenannte **„KANN-Regelung“**, d.h. Sie können bei einer Ethikkommission einreichen, müssen dies aber nicht zwingend tun.

Sollten Sie dennoch Ihr Projekt der Ethikkommission der Stadt Wien zur Begutachtung vorlegen wollen, muss ich Ihnen bedauerlicher Weise mitteilen, dass wir derzeit nicht über die nötigen Ressourcen verfügen, um Studien in Krankenanstalten außerhalb des Wiener Krankenanstaltenverbundes in unserer Kommission zu behandeln.

Mit freundlichen

Grüßen

Der

Geschäftsführer:

Reinhard Undeutsch
