# Supplementary material for: Leaky RAG Deficiency in Adult Patients with Impaired Antibody Production against Bacterial Polysaccharide Antigens
Source: PLoS One. 2015 Jul 17;10(7):e0133220. doi: 10.1371/journal.pone.0133220 (PMC4506145; doi:10.1371/journal.pone.0133220)
Supplement: S1 Table — (DOCX) [file pone.0133220.s004.docx]

| **Name** | **Clone** | **Conjugate** | **Manufacturer** |
| --- | --- | --- | --- |
| CD3 | SK7 | FITC | BD |
| CD4 | MT310 | FITC | Dako |
| CD5 | L17F12 | PE | BD |
| CD8 | DK25 | PE | Dako |
| CD19 | HD37 | FITC | Dako |
| CD20 | L27 | PerCP | BD |
| CD21 | HB5 | PE | BD |
| CD25 | 2A3 | FITC | BD |
| CD27 | O323 | PE | eBiosience |
| CD38 | HIT2 | FITC | BD |
| CD40 | 5C3 | FITC | BD |
| CD45 | T29/33 | FITC | Dako |
| CD56 | C5.9 | PE | Dako |
| CD127 | HIL-7R-M21 | PE | BD |
| CD45RA | L48 | FITC | BD |
| CD45RO | UCHL1 | PE | BD |
| CD62L | DREG-56 | FITC | BD |
| HLA DR | L243 | PE | BD |
| IgD | IA6-2 | FITC | BD |
| IgM | G20-127 | APC | BD |
| TCRVa/b | WT31 | FITC | BD |
