# Supplementary material for: Leaky RAG Deficiency in Adult Patients with Impaired Antibody Production against Bacterial Polysaccharide Antigens
Source: PLoS One. 2015 Jul 17;10(7):e0133220. doi: 10.1371/journal.pone.0133220 (PMC4506145; doi:10.1371/journal.pone.0133220)
Supplement: S2 Table — (DOCX) [file pone.0133220.s005.docx]

| **Gene** |
| --- |
| ACTB |
| ADA |
| ADA |
| ADAM17 |
| AICDA |
| AIRE |
| AK2 |
| AP3B1 |
| APOL-I |
| ATM |
| BLM |
| BLNK |
| BTK |
| C16orf57 |
| C1QA |
| C1QB |
| C1QC |
| C1R |
| C1S |
| C2 |
| C3 |
| C4A |
| C4B |
| C5 |
| C6 |
| C7 |
| C8A |
| C8B |
| C9 |
| CARD11 |
| CARD9 |
| CASP10 |
| CASP8 |
| CD19 |
| CD21 |
| CD247 |
| CD27 |
| CD3D |
| CD3E |
| CD3G |
| CD40 |
| CD40L |
| CD46 |
| CD59 |
| CD70 |
| CD74 |
| CD79A |
| CD79B |
| CD81 |
| CD8A |
| CEBPE |
| CFD |
| CFH |
| CFI |
| CFP |
| CIITA |
| COLEC10 |
| COLEC11 |
| COLEC12 |
| CORONIN1A |
| CSF2RA |
| CTSC |
| CXCR4 |
| CYBA |
| CYBB |
| DCLRE1C |
| DCLRE1C |
| DiGeorge |
| DKC1 |
| DNMT3B |
| DOCK8 |
| ELANE |
| FADD |
| FAS |
| FASLG |
| FCN3 |
| FERMT3 |
| FOXN1 |
| FOXP3 |
| FPR1 |
| G6PC3 |
| GATA2 |
| GFI1 |
| GPD |
| HAX1 |
| ICOS |
| IFNGR1 |
| IFNGR2 |
| IGHM |
| IGLL1 |
| IKBKB |
| IKBKG |
| IKZF1 |
| IL10 |
| IL10RA |
| IL10RB |
| IL12B |
| IL12RB1 |
| IL-17F |
| IL-17RA |
| IL1RN |
| IL2RA |
| IL2RG |
| IL2RG |
| IL36RN |
| IL7R |
| IL7R |
| IRAK2 |
| IRAK4 |
| IRF8 |
| ISG15 |
| ITCH |
| ITGB2 |
| ITK |
| JAK3 |
| LCK |
| LIG1 |
| LIG4 |
| LIG4 |
| LPIN2 |
| LRBA |
| LRRC8A |
| LYST |
| MAGT1 |
| MASP1 |
| MASP2 |
| MBL2 |
| MCM4 |
| MEFV |
| MRE11 |
| MS4A1 |
| MSH5 |
| MSN |
| MVK |
| MyD88 |
| NBN |
| NCF1 |
| NCF2 |
| NCF4 |
| NEMO |
| NFKBIA |
| NHEJ1 |
| NHP2 |
| NKX2-5 |
| NLRP12 |
| NLRP3 |
| NOD2 |
| NOP10 |
| NRAS |
| ORAI1 |
| PIGA |
| PIK3R1 |
| PLCG2 |
| PLDN |
| PMS2 |
| PNP |
| PRF1 |
| PRKCD |
| PRKDC |
| PSTPIP1 |
| PTPRC |
| RAB27A |
| RAC2 |
| RAG1 |
| RAG1 |
| RAG2 |
| RAG2 |
| RFX5 |
| RFXANK |
| RFXAP |
| RHOH |
| RMRP |
| RMRP |
| RNF168 |
| ROBLD3 |
| RPSA |
| SBDS |
| SERPING1 |
| SH2D1A |
| SLC35C1 |
| SLC37A4 |
| SMARCAL1 |
| SP110 |
| SPINK5 |
| STAT1 |
| STAT1 |
| STAT3 |
| STAT5A |
| STAT5B |
| STIM-1 |
| STK4 |
| STX11 |
| STXBP2 |
| TAP1 |
| TAP2 |
| TAPBP |
| TAZ |
| TBK1 |
| TBX1 |
| TERC |
| TERT |
| TINF2 |
| TLR3 |
| TMC6 |
| TMC8 |
| TNFRSF13B |
| TNFRSF13C |
| TNFRSF1A |
| TRAC |
| TRAF3 |
| TRIF |
| TYK2 |
| UNC119 |
| UNC13D |
| UNC93B1 |
| UNG |
| VPS13B |
| WAS |
| WIPF1 |
| XIAP |
| ZAP70 |
| ZBTB24 |
