# Supplementary material for: Leaky RAG Deficiency in Adult Patients with Impaired Antibody Production against Bacterial Polysaccharide Antigens
Source: PLoS One. 2015 Jul 17;10(7):e0133220. doi: 10.1371/journal.pone.0133220 (PMC4506145; doi:10.1371/journal.pone.0133220)
Supplement: S3 Table — (DOCX) [file pone.0133220.s006.docx]

| **Name** | **Sequence** |
| --- | --- |
| HuBV1 | CCGCACAACAGTTCCCTGACTTGC |
| HuBV2 | CACAACTATGTTTTGGTATCGTC |
| HuBV3 | CGCTTCTCCCTGATTCTGGAGTCC |
| HuBV4 | TTCCCATCAGCCGCCCAAACCTAA |
| HuBV5 | GATCAAAACGAGAGGACAGC |
| HuBV6A | GATCCAATTTCAGGTCATACTG |
| HuBV6B | CAGGGSCCAGAGTTTCTGAC |
| HuBV6C | CAGGGCTCAGAGGTTCTGAC |
| HuBV7 | CCTGAATGCCCCAACAGCTCT |
| HuBV8 | GGTACAGACAGACCATGATGC |
| HuBV9 | TTCCCTGGAGCTTGGTGACTCTGC |
| HuBV11 | GTCAACAGTCTCCAGAATAAGG |
| HuBV12 | TCCYCCTCACTCTGGAGTC |
| HuBV13A | GCATGACACTGCAGTGTGCCC |
| HuBV13B | AGGCTCATCCATTATTCAAATAC |
| HuBV14 | GGGCTGGGCTTAAGGCAGATCTAC |
| HuBV15 | CAGGCACAGGCTAAATTCTCCCTG |
| HuBV16 | GCCTGCAGAACTGGAGGATTCTGG |
| HuBV17 | TCCTCTCACTGTGACATCGGCCCA |
| HuBV18 | CTGCTGAATTTCCCAAAGAGGGCC |
| HuBV20 | TGCCCCAGAATCTCTCAGCCTCCA |
| HuBV21 | GGAGTAGACTCCACTCTCAAG |
| HuBV22 | GATCCGGTCCACAAAGCTGG |
| HuBV23 | ATTCTGAACTGAACATGAGCTCCT |
| HuBC1 | GGGTGTGGGAGATCTCTGC |
| HuBC1-FAM | FAM-TTGGGTGTGGGAGATCTCTGC |
| qRT_RAG1_f | GATCTCACCCGGAACAGCTT |
| qRT_RAG1_r | AGCGCAAGAGAAGAGCTCAG |
| qRT_HPRT_f | GCTATAAATTCTTTGCTGACCTGCTG |
| qRT_HPRT_r | AATTACTTTTATGTCCCCTGTTGACTGG |
| sspRAG1-A1w_f | CTCAGGTAC CTCAGCCACCA |
| sspRAG1-G1w_f | CTCAGGTACCTCAGCCACCG |
| gRAG1E2-2seq_r | ACCCCTTTCACCCGATCCCGCAGT |
| sspRAG1-G2210w_r | CAAGATTTTGAGAGGCTTCCACAC |
| sspRAG1_A2210m_r | CAAGATTTTGAGAGGCTTCCACAC |
| gRAG1E2-1_f | ACTGGGACCCTTGGGGAGGCAAAGA |
| gRAG2E2-2seq_f | AGACCCCAGATTGGACCCCAGAC |
| gRAG2E2-2seq2_r | ACTGCTTCTGGGTGTTTAAGGTTTGTTGA |
| RAG2E2-2_f | CCTGCCCCACTGGAGTTTTCCATCTGG |
| gRAG2E2-2seq1_r | AGGGAGTGGAATCCCCTGGATCTTCT |
